# Insights into life after sport for Spanish Olympians: Gender and career path perspectives

**DOI:** 10.1371/journal.pone.0209433

**Published:** 2018-12-17

**Authors:** Maribel Barriopedro, Cristina López de Subijana, Carlos Muniesa

**Affiliations:** Department of Social Sciences Applied to Sport, Physical Activity and Leisure, Sport Sciences Faculty, Universidad Politécnica de Madrid, Madrid, Spain; Hochschule Trier, GERMANY

## Abstract

The aims of this study were: i) to examine if Dual Career (DC) pathways are independent of gender, ii) to evaluate whether those athletes who followed a DC experienced less difficulty in their integration into the labor force than the athletes devoted entirely to sport regardless of gender, as well as iii) to analyze whether the type of career path chosen was related with the current labor status, and if differences exist between men and women athletes. A quantitative, cross-sectional, and descriptive study was used based on an ad hoc questionnaire. Two-hundred and twenty-eight retired Olympic athletes completed a questionnaire. The response rate was 28.3%. Athletes who followed a DC with studies had a higher educational level at retirement than those devoted solely to sport and those who followed a DC with work, (χ^2^(6) = 38.76; *P* < .001), but no differences were found between men and women (χ^2^(3) = 3.23; *P* = .358). Athletes who followed a DC path (with studies or with work) perceived the transition out of sport more positively than those who focused solely on sport (*χ*^*2*^(2) = 7.79; *P* = .020). Regarding the type of job, more women attained a part-time job (20.9%) than men (3.1%; *χ*^*2*^(5) = 21.83; *P* = .001). The athletes who followed a DC with studies achieved higher monthly incomes than the other two groups (*χ*^*2*^(2) = 9.08; *P* = .011). Men athletes achieved higher incomes than women (Z = 5.45; *P* < .001), but the gender wage gap was apparent for those Olympian athletes considered as the qualified group, probably due to a higher presence of part-time women workers. The findings of this study suggest that future professional opportunities and the transition to the labor market could be made easier by following a DC during the mastery stage. Regardless of career path, women experienced more difficulties in their integration into the job market and there is a wage penalty for highly-qualified women.

## Introduction

The European Union coined the term “dual career” (DC) to define the career that an elite athlete has in terms of studying and/or working while at the same time pursuing high-performance sport [1]. The pillar of this concept is based on the Holistic Athletic Career model proposed by Wylleman, Reints and De Knop [2]. This model addresses the individual’s development across different domains (athletic, psychological, psychosocial, academic/vocational, and financial) and provides a description of the transitions that athletes may face throughout their athletic lifespan. The underlying premise of this model implies that there are interactions between all the development domains [3].

Athletes choose their career paths, making a decision between being solely devoted to sport or following a DC. According to the British Association of Universities and Colleges Sport (BUCS), 61% of the UK Olympic athletes between the 1992 Barcelona Olympics to the 2012 London Olympics were student-athletes [4]. In France, more than half of the medalists in the 2000 Olympic Games in Athens were at France’s National Institute of Sport [5]. In Finland, 65% of the Olympic athletes were also students [6]. In Spain, a survey of retired Olympic athletes demonstrated that 51% studied and 17% worked during their mastery stage [7]. Based on previous research [7–9] the support programs recommend following a DC, as being focused on just one single activity may lead to problems in the psychological and psychosocial domains. A recent study in the United Kingdom highlighted the risk of developing a one-dimensional identity being solely devoted to sport [10]. The study analyzed the lifespan career development of elite and professional athletes, identified through UK Sport’s elite sport program utilizing narrative methodology. In survey research conducted in Australia [11], as well as in a longitudinal study with semi-structured interviews of 15 athletes with a 10-year interval developed in Spain [12], athletes reported that a DC path helped them to achieve a sense of well-being and a well-rounded life during their mastery stage.

When comparing the retirement transitions of the athletes devoted entirely to sport with those of the athletes who followed a DC, the athletes that combined sport with another activity demonstrated having fewer difficulties and a better integration into the labor market [6, 12]. However pursuing goals outside of sport in the same period as big events is complicated to manage [13–15]. The second activity of the DC could be studies or work. Some researchers have pointed out that these student-athletes do not perform well academically [16–18]. More recently, other studies have suggested that the level of education achieved by student-athletes is even better than the general population [8,19,20], despite these students take more time to finish their degrees [21]. Conversely, at work, the competition schedule of traveling and training is difficult for both workers (athletes) and employers [14].

Taking gender into consideration, some inequalities appear. Following the Education at a Glance 2017 report of the Organization for Economic Co-operation and Development (OECD) [22] although women tend to be generally the disadvantaged group in society in most countries, the reverse is true when analyzing education data. These data display gender gaps in favor of women, such as completion rate in upper secondary education and participation and completion in tertiary education, even though they are paid less than their counterparts. According to Eurostat [23] the pay or wage gap in Spain is 14.9% while in the European Union it is 16.3%.

Previous studies have not addressed the current labor status of the retired athletes and most of them have not taken gender into account, therefore the aims of this study were: i) to examine if DC pathways are independent of gender, ii) to evaluate whether those athletes who followed a DC experienced less difficulty in their labor market integration than the athletes devoted entirely to sport regardless of gender, as well as iii) to analyze whether the type of career path chosen was related with the current labor status, and if differences exist between men and women athletes.

## Methods

### Participants

The target population was the Spanish Olympic athletes who participated in the summer Olympic Games of Barcelona 1992, Atlanta 1996, or Sydney 2000. From the initial population of 1052 participants in the three above mentioned Olympic Games, the deceased (2) or those who repeated their participation (245) were excluded. The final population for this research was 805. The questionnaires were sent by surface mail to the athlete’s personal address during three consecutive years. The final sample included 228 athletes (57.9% men and 42.1% women), representing an overall response rate of 28.3%. Assuming the most disadvantageous situation (*p = q*), and with a confidence interval of 95%, the overall estimation error was ± 5.5%. Participants had an average age of 39.3 ± 5.0 years. In relation with their sport performance in the Olympic Games, 18.8% of the sample obtained a medal while 29% were awarded a diploma. They had been retired for a minimum of five years before the administration of the questionnaire. The Spanish Olympic Committee ethics committee approved the present protocol.

### Measures

The questionnaire was developed based on different sources. Firstly, the National Labor Force Survey was taken into account. Secondly, a panel of eight experts with different skills and experience (sport sciences research, the Olympic athlete, the elite athlete and the sport law in Spain) had several meetings to draw up the first version of the questionnaire. The panel of experts was composed of members of the Spanish National Olympic Committee (Athletes Commission and the Executive Committee) and from the Universidad Politécnica de Madrid (Department of Social Sciences applied to Sport). All the experts selected had a minimum of 15 years experience in their field.

In order to assess the suitability and understanding of the questions, a pilot study was carried out with 15 athletes. The final version of the questionnaire was called the Social and Working Integration Questionnaire [24]. It had 42 questions distributed into five sections: sociodemographic characteristics, sport profile, academic profile, work status, and retirement from sport.

Many of the measures included in our study were factual questions (i.e. career path, educational level, how much time they took to find the first job, income per month). The information required in those factual questions were difficult to reach by other methods [25, 26]. Nevertheless, the congruency among different factual questions (i.e. career path and educational level reached at retirement) gives us some evidence about their validity. On the other hand, in order to maximize the accuracy of answers, it was important to guaranty the anonymity, so subjects cannot be reached twice. Then, test retest reliability for each questions, was not suitable for this kind of study [25].

The sociodemographic characteristics section includes age, sex, marital status, and family size. The sport profile section includes five questions, related to the Olympic Games and sports in which the athletes participated, athletic achievements, if they attained or not an ADO (Ayuda a Deportistas Olímpicos [Aid for Olympic Athletes]) scholarship and career path followed during their mastery stage. The academic profile section includes nine questions, about their level of education achieved at retirement and at the time of the study, parents’ educational level, if the studies were related with sport, time, support and difficulties to accomplish studies. The work status section includes 13 questions to gain information about the following focal areas: employment status, monthly income, if the job is related to sports, strategies to find the first job and living conditions. Finally, the retirement from sport section includes 11 questions to obtain data on reasons for sports career termination, the gradualness of the sport career termination, how difficult they perceived their integration into the labor market after the sport career, the time they took until they found their first job, access to programs designed for retiring elite athletes and their physical activity and sport practice after retirement and nowadays.

The questions related to the objectives of this study were selected. Athletes were asked to recall their career path during their mastery stage (the stage at which athletes reach their highest level of sport performance) and they had to choose from a multiple choice question: a) I was solely devoted to sport; b) I combined sport and education and c) I combined sport and a working activity. To verify whether those athletes who followed a DC based on sport and studies finished their degrees, the athletes answered about their level of education achieved at retirement and at the time of the study. Following the International Standard Classification of Education [27], we classified the participants’ educational level as (i) Primary Education (common education organized into six years that students follow between the ages of 6 and 12), (ii) Secondary Education (preparing for tertiary education. It is organized into six years that students follow between the ages of 12 and 18), (iii) Professional training (2 to 5 year programs that are typically practical-based, occupationally-specific and prepare for labor market entry. These programs may also provide a pathway to other tertiary courses when their duration is 5 years) and (iv) Tertiary Education (university-level degree equivalent to 3 or 5 years of university education, Bachelor, Master or Doctorate).

To analyze whether the type of the chosen career path was related to difficulties experienced with regard to integration into the labor market and with the current job, four more questions were selected. In particular, three of the questions were objective, as they had to report their monthly income from different ranges at the time of the study, to acknowledge the time they took until they found their first job and to choose their professional situation at the time of the study from a multiple choice question. The options given for this question were: being employed (full-time, part-time or temporarily), being an entrepreneur, professional or self-employed. Conversely, the fourth question was subjective, and it asked how difficult they perceived their integration into the labour market after the sport career on a Likert scale ranging from 1 (i.e., “very badly”) to a maximum of 6 (i.e., “very well”).

### Procedure

This research was a quantitative, cross-sectional, and descriptive survey study based on a questionnaire that had been sent by the NOC to the entire aforementioned population by surface mail. To increase the sample size, the NOC submitted the questionnaires a second time. Those athletes that already answered the questionnaire did not participate in the next sampling. Prior to completing the questionnaire, the athletes signed a consent form; however, in order to keep the anonymity of participants, the NOC coded the questionnaires upon their arrival.

### Data analysis

Responses were coded and entered into Predictive Analytics Software, v. 21. Non-parametric statistics (Kruskall-Wallis, Mann-Witney U or a Pearson Chi Square test) were applied to compare the differences between groups. The *η*^*2*^ index and Cramer’s *V* (C_v_) coefficient were the effect size indicators, and, in line with other studies [28], *η*^*2*^ = 0.01 and *C*_*v*_ = 0.10, *η*^*2*^ = 0.06 and *C*_*v*_ = 0.30, *η*^*2*^ = 0.14 and *C*_*v*_ = 0.50 were considered as low, medium, and large effect sizes, respectively. A classification tree analysis was performed for those dependent variables simultaneously related with career path and gender. This technique allows splitting the sample into subgroups (nodes) based on the impact of each independent variable. The algorithm used was the exhaustive CHAID (Chi-squared Automatic Interaction Detection). The Chi-Squared test identifies the relationships between independent variables through completing three steps on each node of the root (merging, splitting and stopping) to find the predictors that exert the most influence on the dependent variable [29]. The following statistical specifications were considered: i) significance level was set at .05; ii) the maximum number of interactions was 100; iii) the minimum change in expected cell frequencies was .001; iv) the significant values adjustment was performed using the Bonferroni method; and v) the tree had a maximum of 3 levels. Finally, the risk of misclassification was calculated as a measure of the reliability of the model.

## Results

### Dual career and educational level

Most of the athletes (66.2%) combined studies and sport while they were at the mastery stage of their sport career (Table 1), regardless of gender (χ^2^(2) = 1.08; *P* = .584). Only 9.6% of them followed a DC with work.

**Table 1:** Career path chosen by gender.

	Solely devoted to sport (%)	Dual Career with studies (%)	Dual Career with work (%)
**Men (N = 132)**	26.5	63.6	9.8
**Women (N = 96)**	20.8	69.8	9.4
**Total (N = 228)**	24.1	66.2	9.6

Table 2 shows the level of education at retirement and at the moment of the study by career path and by gender. Significant differences were found regarding the educational level reached at retirement according to the career path chosen (χ^2^(6) = 38.76; *P*< .001; *C*_*v*_ = .292) but no differences were found between men and women (χ^2^(3) = 3.23; *P* = .358). While 53.3% of the athletes who followed a DC with studies had finished university (Tertiary Education) at the time of retirement, only 14.5% of those devoted solely to sport and 27.3% of those who followed a DC with work had achieved that educational level.

**Table 2:** Educational level by career path and gender.

	Solely devoted to sport	Dual Career with studies	Dual Career with work	Men	Women
	% (N = 55)	% (N = 150)	% (N = 22)	% (N = 132)	% (N = 95)
**Education at retirement**					
** Primary Education**	27.3	5.3	13.6	9.8	13.7
** Professional training**	16.4	6.7	18.2	12.9	6.3
** Secondary Education**	41.8	34.7	40.9	35.6	38.9
** Tertiary Education**	14.5	53.3	27.3	41.7	41.1
**Education Nowadays**					
** Primary Education**	21.8	0.7	9.1	8.3	4.2
** Professional training**	18.2	3.3	13.6	9.8	5.3
** Secondary Education**	32.7	10.7	31.8	18.2	17.9
** Tertiary Education**	27.3	85.3	45.5	63.6	72.6

Significant differences were found regarding the educational level reached at the moment of the study according to the career path chosen (χ^2^(6) = 74.9; *P* < .001; *C*_*v*_ = .406) but no differences were found between men and women (χ^2^(3) = 3.55; *P* = .314). The athletes who followed a DC with studies achieved a higher level of education than those who were focused solely on sport and those who followed a DC with work.

### Dual career and labor market integration

**Fig 1:**
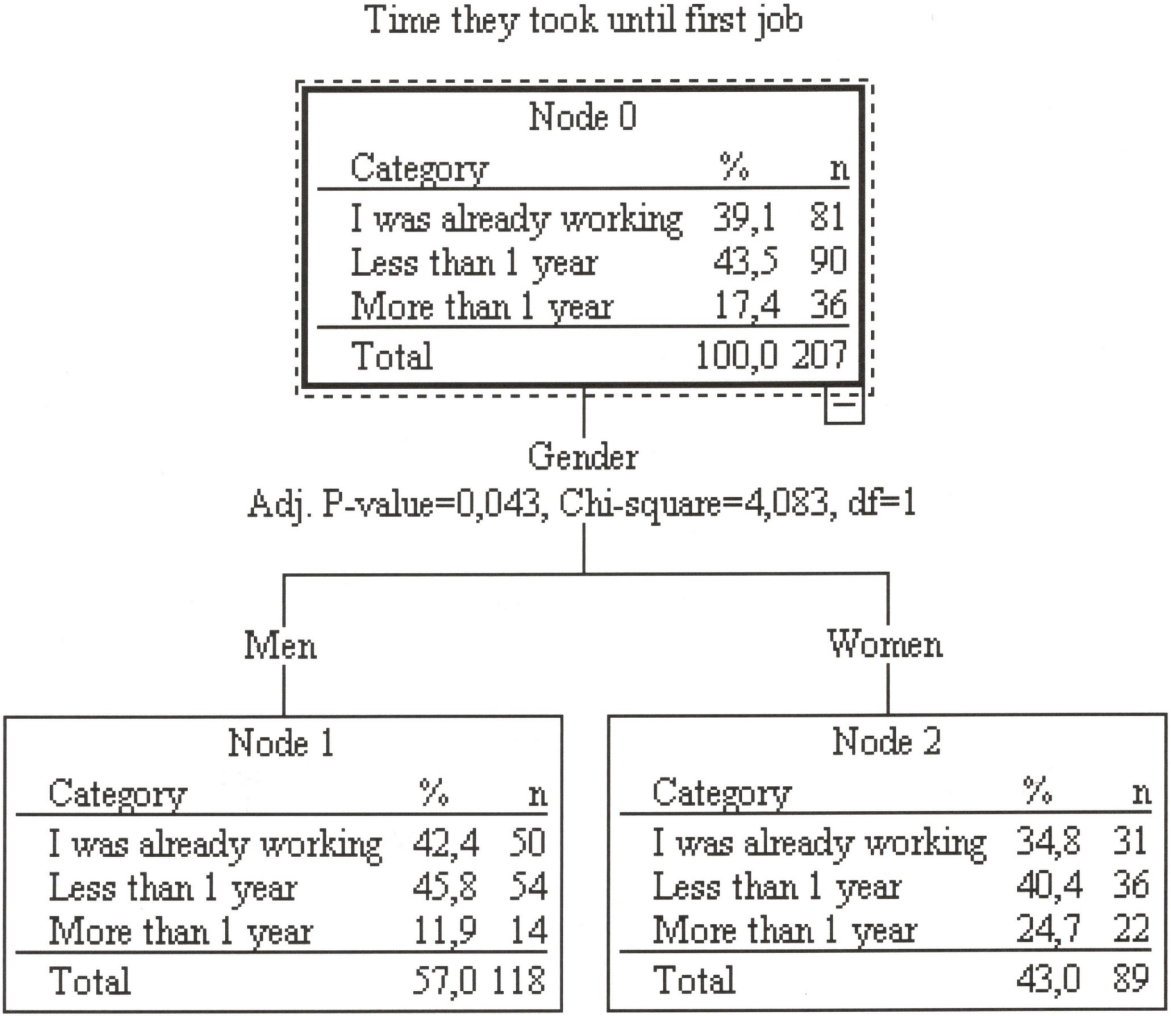
Classification tree analysis of time needed to get their first job.

The time necessary for athletes to get their first job differed among the three groups (*χ*^*2*^(4) = 24.2; *P* < .001; *C*_*v*_ = .242) (Table 3). The athletes that followed a DC with studies or work were already working more frequently than those focused on sport (83.3% for those that combined sport and working; 40.0% for those that combined sport and studies and 20.4% for those devoted to sport). More women (24.7%) than men (11.9%) athletes took more than one year to get their first job (*χ*^*2*^(1) = 5.89; *P* = .05; *C*_*v*_ = .169). The classification tree showed only gender as a significant factor for predicting the time necessary to get the first job (Fig 1). This classification model was able to correctly classify 43.5% of the cases.

**Table 3:** Time needed to get the first job by career path and gender.

	Solely devoted to sport	Dual Career with studies	Dual Career with work	Men	Women
	% (N = 49)	% (N = 140)	% (N = 18)	% (N = 118)	%(N = 89)
**I was already working**	20.4	40.0	83.3	42.4	34.8
**Less than 1 year**	63.3	40.7	11.1	45.8	40.4
**More than 1 year**	16.3	19.3	5.6	11.9	24.7

When the athletes were asked how they perceived their labor market integration on a scale from 1 = very badly to 6 = very well, the career path reported a difference (*χ*^*2*^(2) = 7.79; *P* = .020; *η*^*2*^ = .035). In fact, the athletes who followed a DC path (with studies or with work as a second activity; both groups) perceived the transition from their sport career to retiring more positively (4.2 ± 1.6 for DC with studies and 4.1 ± 1.6 for DC with work) than those who focused solely on sport (3.5 ± 1.5; *P* < .05 for both comparisons). No differences were found between men (4 ± 1.7) and women (4 ± 1; *Z* = 0.16; *P* = .872).

### Dual career and professional situation

The professional situation of the athletes (Table 4) did not differ in relation to the career path (*χ*^*2*^(10) = 6.46; *P* = .775) but gender differences were found (*χ*^*2*^(5) = 21.83; *P* = .001; *Cv* = .320). More women athletes got a part-time job (20.9%) than men athletes (3.1%).

**Table 4:** Professional situation and monthly income by career path and gender.

	Solely devoted to sport	Dual Career with studies	Dual Career with work	Men	Women
	% (N = 53)	% (N = 139)	% (N = 21)	% (N = 127)	% (N = 86)
**Professional situation**					
** Full- time job**	60.4	62.6	57.1	69.3	50.0
** Part-time job**	13.2	8.6	14.3	3.1	20.9
** Temporary job**	7.5	4.3	4.8	5.5	4.7
** Entrepreneur**	7.5	9.4	4.8	9.4	7.0
** Professional**		5.0	9.5	2.4	7.0
** Self-employed**	11.3	10.1	9.5	10.2	10.5
**Monthly Income**					
** Up to 600€**	2.0	2.1	4.5	2.3	2.3
** From 600 to 1000€**	15.7	7.0	9.1	5.5	14.8
** From 1000 to 1500€**	23.5	18.9	27.3	13.3	31.8
** From 1500 to 2000€**	29.4	21.7	27.3	19.5	30.7
** From 2000 to 3000€**	21.6	28.0	22.7	34.4	13.6
** More than 3000€**	7.8	22.4	9.1	25.0	6.8

The income level of the athletes (Table 4) varied in relation to the career path (*χ*^*2*^(2) = 9.08; *P* = .011; *η*^*2*^ = .042). The athletes who followed a DC with studies achieved higher monthly incomes than those who were focused solely on sport and those who combined work and sport (*P* < .05 for both comparisons). No significant differences were found regarding the income level between these last two groups (*P* >.05). Men athletes achieved higher incomes than women (*Z* = 5.45; *P*< .001; *η*^*2*^ = .025).

**Fig 2:**
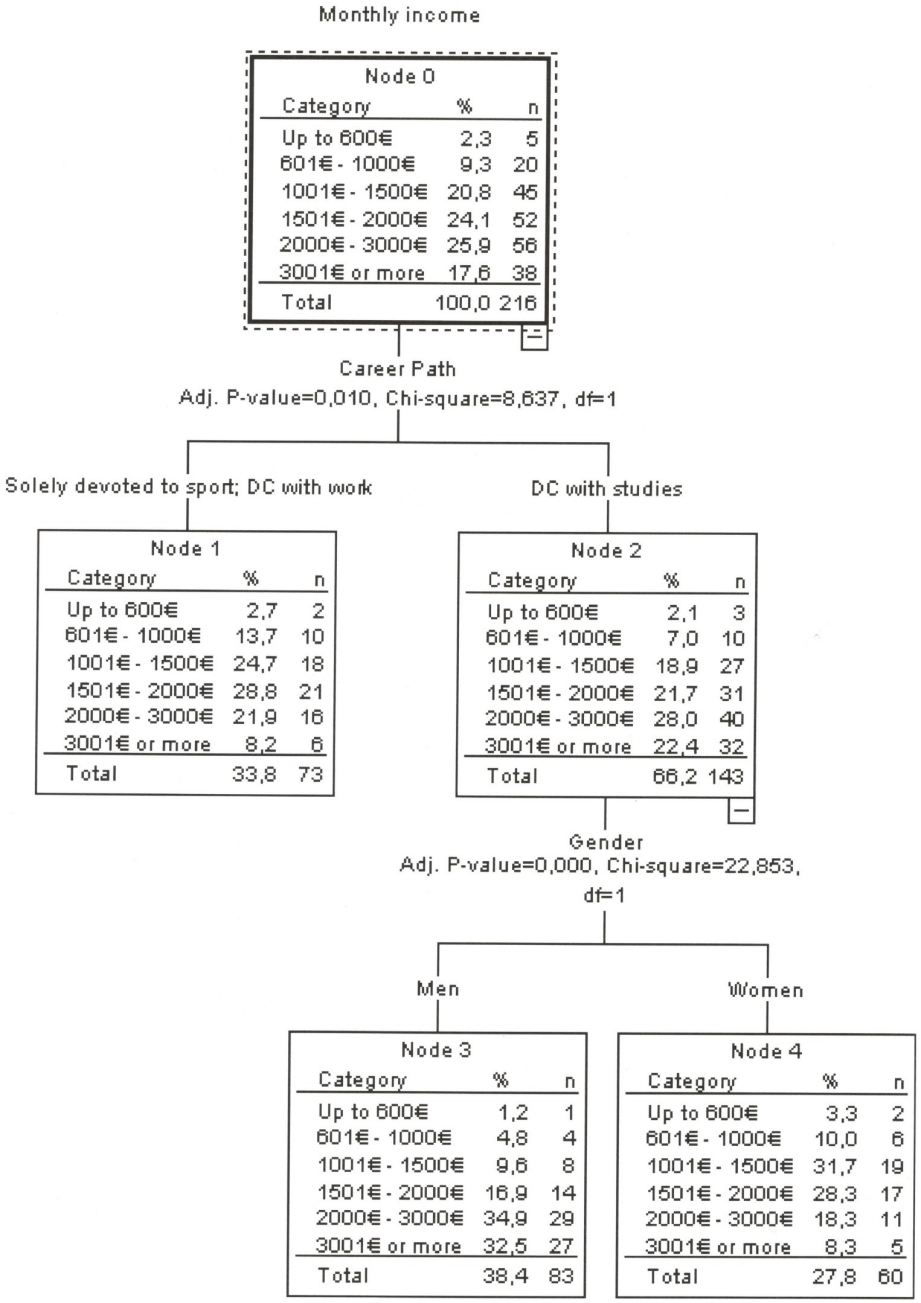
Classification tree analysis of monthly income.

The classification tree included career path and gender as significant factors in the statistical model. Higher monthly incomes were predicted for athletes who followed a DC with studies and only among them, higher monthly incomes were predicted for men athletes (Fig 2). This classification model correctly classified 31.9% of the cases.

## Discussion

The aim of the present study was to examine if DC pathways and the advantages associated with a DC in relation to integration into the labor market were independent of gender. No relationship emerged between gender and career path. This study shows how following a DC during the mastery stage contributes positively to facilitating integration into the labor market at the end of the sport career. The results support the importance of a holistic view of athletic career development [2] reflecting an interrelation of the athletic and academic/vocational domains, and points out the possible role of gender in career development.

### Dual career and level of education

The distribution of the athletes’ career paths within the sample are in line with previous studies on the Spanish athlete population [7,12], and are similar to the number of student-athletes in other countries like the UK (61%) or Finland (65%) [4,6].

The commitment acquired during the mastery stage by those athletes who followed a DC with studies is shown with the higher levels of education achieved at retirement than those who followed a DC with work or were focused solely on sport. Furthermore, a large number of those athletes decided to continue their academic career after retirement. In contrast with the findings of Tekavc et al. [14] women athletes from our study did not achieve higher levels of education than men. For this population (Spanish Olympic athletes), the reversed gender gap in education that holds for almost all European countries, was not confirmed [22].

### Dual career and labor market integration

The results showed how the combination of sport training and education/work activity during the sport career contributes positively to facilitating the retirement process, confirming the theses of previous studies [11,12,15]. In particular, those athletes that follow a DC (with studies or work) attain their first job earlier and perceive their transition to the labor market more positively than the athletes devoted solely to sport. However, only gender contributes significantly to explain differences in the time needed to attain the first job when gender and career path are taken simultaneously into account.

Many of the athletes who combined sport and education simultaneously had attained a university degree. As educational status is a key issue in finding suitable employment, the elite athletes who obtain a higher education degree, have fewer problems finding employment once their sport career is finished [7,12] On the other hand, it is assumed that athletes who combined sport and work acquired previous vocational training, which would be particularly valued in the labor market. Since the percentages of women and men than reached a higher education degree at retirement or that followed a DC with work are similar, the differences in the time needed to attain the first job might reflect unequal employment conditions.

### Dual career and professional situation

Our results point out that elite athletes who followed a DC with studies attain higher monthly incomes than the other two career paths. Although there is concern in the European Commission regarding the insertion of elite athletes into the labor market [30] the athletes from this sample attained higher incomes than the general population of the same age [31]. These results are in agreement with those found in Germany, where the Olympic athletes’ economic status resulted in being higher than the general population [19]. In fact, the Olympic level is the highest level that an athlete could achieve in some sports. Then the achievement of some general life skills and social status while they pursue their sport goal are easily speculated. Those advantages are then taken into account when finding a job because Olympic athletes are better positioned in comparison with the general population [19, 32]. Only athletes who followed a DC with studies showed the wage gap associated with gender. Although gender discrimination was found to be a less serious problem for the most highly educated women, there is a wage penalty associated with part-time jobs, especially for highly-qualified women [33]. It is important to remark that in our findings among those that had followed a DC with studies, more women (18.6%) than men (1.3%) had part-time jobs.

### Strengths and limitations

Although this study presents valuable information from a large sample with a retrospective point of view, some limitations should be pointed out. First, due to the low response rate, the lack of knowledge of those athletes who did not answer the questionnaire could bias the results. In fact, it could be presumed that the athletes who had a bad retirement experience may have been reluctant to participate in the study. Although this represent a severe limitation on external validity, differences between career paths and gender still provides important information. Second, there is a risk of memory decay and recall bias associated with retrospective designs. Finally, this study did not take into account the changes in the career path during the mastery stage or how athletes that follow a DC combine the two pursuits (sport and education or work). This means that we did not gather detailed information as to the number of years in each career path or number or hours of courses per year that may have enriched the study. Nevertheless, it seems that at least with the global perception of the athletes during that life stage it is possible to discriminate employment and financial status nowadays.

Many athletes that follow a DC with studies report sport as the pursuit they prioritized. Such prioritizing of sport at the expense of education is frequent in the context of the Spanish sport system where the financial support is based exclusively on sport results. Knowing the importance of a balanced DC for the athletes’ wellbeing, in 2007 the Spanish government developed and approved Royal Decree 971 on elite athletes that included measures to facilitate a DC. Future research might explore if those legal measures facilitated, to a greater degree, future professional opportunities and the transition to the labor market.

### Conclusions

The findings of this study suggest that the professional future opportunities and the transition to the labor market could be easier by following a DC during the mastery stage. Choosing to be solely devoted to one’s sport career could be seen as acting against long-term financial and employment interests [30]. The efforts of the Spanish stakeholders to promote education among the athletes are reflected in the vanishing reverse education gender gap for Olympic athletes. However, a significant level of discrimination against women is found among these Olympic athletes. Compared to women, men got their first job earlier and more women than men had part-time jobs. Furthermore, there is a wage penalty, especially for highly-qualified women. These differences are in line with those reported nowadays in the Spanish labor market.

Therefore, supporting programs should continue working on increasing the rate of athletes that follow a DC while they are doing their best in sport. In that way, the athletes will be better prepared for their life after sport. Gender should also be taken into account in order to promote equality between men and women [34].

## Supporting information

S1 File The data base of the research. (PDF)
