# Supplementary material for: Insights into life after sport for Spanish Olympians: Gender and career path perspectives
Source: PLoS One. 2018 Dec 17;13(12):e0209433. doi: 10.1371/journal.pone.0209433 (PMC6296509; doi:10.1371/journal.pone.0209433)
Supplement: S1 File — (PDF) [file pone.0209433.s001.pdf]

ID	Gender	YearBirth	CareerPath	MonthlyIncome	BestResult	Difficult_Integration	Time_First_Job	Education_Retirement	Actual_Education	Professional_Situation
1	Men	1.968	Solely devoted to sport	1501€ - 2000€	Other result	Very well	Less than 1 year	Professional Training	Professional Training	Full- time job
2	Men	1.971	Solely devoted to sport	#¡NULO!	Gold	Very well	#¡NULO!	Secondary Education	Secondary Education	Temporary job
3	Men	1.968	Solely devoted to sport	#¡NULO!	Diploma	Very bad	Less than 1 year	Secondary Education	Secondary Education	Entrepreneur
4	Men	1.972	Solely devoted to sport	1501€ - 2000€	Other result	2	Less than 1 year	Primary Education	Primary Education	Self-employed
5	Men	1.966	Solely devoted to sport	#¡NULO!	Other result	4	Less than 1 year	Professional Training	Professional Training	Full- time job
6	Men	1.968	Solely devoted to sport	3001€ or more	Other result	3	Less than 1 year	Secondary Education	Secondary Education	Full- time job
7	Men	1.966	Solely devoted to sport	2000€ - 3000€	Bronze	2	Less than 1 year	Secondary Education	Tertiary Education	Self-employed
8	Men	1.968	Solely devoted to sport	1501€ - 2000€	Other result	3	More than 1 year	Secondary Education	Tertiary Education	Full- time job
9	Men	1.969	Solely devoted to sport	2000€ - 3000€	Other result	4	Less than 1 year	Primary Education	Primary Education	Full- time job
10	Men	1.970	Solely devoted to sport	1001€ - 1500€	Diploma	Very bad	I was already working	Primary Education	Primary Education	Full- time job
11	Men	1.968	Solely devoted to sport	1501€ - 2000€	Silver	2	I was already working	Secondary Education	Tertiary Education	Self-employed
12	Men	1.959	Solely devoted to sport	2000€ - 3000€	Silver	2	I was already working	Tertiary Education	Tertiary Education	Full- time job
13	Men	1.965	Solely devoted to sport	2000€ - 3000€	Other result	Very well	Less than 1 year	Tertiary Education	Tertiary Education	Full- time job
14	Men	1.961	Solely devoted to sport	1501€ - 2000€	Diploma	3	Less than 1 year	Tertiary Education	Tertiary Education	Full- time job
15	Men	1.965	Solely devoted to sport	601€ - 1000€	Diploma	4	#¡NULO!	Primary Education	Primary Education	Temporary job
16	Men	1.968	Solely devoted to sport	1001€ - 1500€	Diploma	5	Less than 1 year	Secondary Education	Secondary Education	Full- time job
17	Men	1.969	Solely devoted to sport	1501€ - 2000€	Gold	4	I was already working	Primary Education	Primary Education	Full- time job
18	Men	1.969	Solely devoted to sport	1501€ - 2000€	Diploma	3	More than 1 year	Secondary Education	Secondary Education	Full- time job
19	Men	1.974	Solely devoted to sport	1501€ - 2000€	Gold	Very well	Less than 1 year	Secondary Education	Secondary Education	Full- time job
20	Men	1.968	Solely devoted to sport	1501€ - 2000€	Other result	Very well	Less than 1 year	Professional Training	Professional Training	Full- time job
21	Men	1.970	Solely devoted to sport	1001€ - 1500€	Other result	2	Less than 1 year	Primary Education	Professional Training	#¡NULO!
22	Men	1.969	Solely devoted to sport	2000€ - 3000€	Other result	4	Less than 1 year	Primary Education	Primary Education	Full- time job
23	Men	1.970	Solely devoted to sport	1001€ - 1500€	Bronze	3	#¡NULO!	Primary Education	Primary Education	Temporary job
24	Men	1.966	Solely devoted to sport	2000€ - 3000€	Bronze	2	Less than 1 year	Secondary Education	Tertiary Education	Self-employed
25	Men	1.968	Solely devoted to sport	3001€ or more	Other result	Very well	More than 1 year	Secondary Education	Secondary Education	Full- time job
26	Men	1.973	Solely devoted to sport	2000€ - 3000€	Diploma	5	I was already working	Secondary Education	Secondary Education	Full- time job
27	Men	1.971	Solely devoted to sport	2000€ - 3000€	Other result	5	Less than 1 year	Professional Training	Professional Training	Full- time job
28	Men	1.970	Solely devoted to sport	2000€ - 3000€	Other result	3	Less than 1 year	Professional Training	Professional Training	Full- time job
29	Men	1.977	Solely devoted to sport	Up to 600€	Other result	2	Less than 1 year	Professional Training	Professional Training	Entrepreneur
30	Men	1.976	Solely devoted to sport	1501€ - 2000€	Other result	5	#¡NULO!	Secondary Education	Secondary Education	Temporary job
31	Men	1.968	Solely devoted to sport	2000€ - 3000€	Bronze	4	Less than 1 year	Professional Training	Professional Training	Entrepreneur
32	Men	1.966	Solely devoted to sport	1001€ - 1500€	Other result	3	Less than 1 year	Tertiary Education	Tertiary Education	Self-employed
33	Men	#¡NULO!	Solely devoted to sport	2000€ - 3000€	Other result	2	Less than 1 year	Professional Training	Professional Training	Full- time job
34	Men	1.969	Solely devoted to sport	3001€ or more	Bronze	5	#¡NULO!	Primary Education	Primary Education	Full- time job
35	Men	1.970	Solely devoted to sport	601€ - 1000€	Other result	Very bad	More than 1 year	Professional Training	Professional Training	Full- time job
36	Men	1.966	DC with studies	2000€ - 3000€	Other result	3	Less than 1 year	Secondary Education	Tertiary Education	Full- time job
37	Men	1.963	DC with studies	3001€ or more	Other result	Very well	I was already working	Tertiary Education	Tertiary Education	Full- time job
38	Men	1.965	DC with studies	1001€ - 1500€	Diploma	Very bad	Less than 1 year	Secondary Education	Tertiary Education	Full- time job
39	Men	1.966	DC with studies	2000€ - 3000€	Other result	Very well	Less than 1 year	Tertiary Education	Tertiary Education	Entrepreneur
40	Men	1.959	DC with studies	3001€ or more	Diploma	Very well	I was already working	Tertiary Education	Tertiary Education	Self-employed
41	Men	1.962	DC with studies	3001€ or more	Diploma	Very well	Less than 1 year	Tertiary Education	Tertiary Education	Entrepreneur
42	Men	1.964	DC with studies	1501€ - 2000€	Diploma	Very well	I was already working	Tertiary Education	Tertiary Education	Full- time job
43	Men	1.970	DC with studies	2000€ - 3000€	Other result	3	Less than 1 year	Secondary Education	Tertiary Education	Full- time job
44	Men	#¡NULO!	DC with studies	2000€ - 3000€	Gold	2	Less than 1 year	Tertiary Education	Tertiary Education	Full- time job
45	Men	1.969	DC with studies	1001€ - 1500€	Other result	Very well	I was already working	Secondary Education	Secondary Education	Full- time job
46	Men	1.963	DC with studies	2000€ - 3000€	Diploma	2	I was already working	Tertiary Education	Tertiary Education	Self-employed
47	Men	1.969	DC with studies	2000€ - 3000€	Other result	Very well	More than 1 year	Tertiary Education	Tertiary Education	Full- time job
48	Men	1.964	DC with studies	1001€ - 1500€	Diploma	5	Less than 1 year	Tertiary Education	Tertiary Education	Full- time job
49	Men	1.960	DC with studies	3001€ or more	Other result	5	I was already working	Tertiary Education	Tertiary Education	Self-employed
50	Men	1.963	DC with studies	2000€ - 3000€	Other result	Very well	Less than 1 year	Professional Training	Tertiary Education	Full- time job
51	Men	1.968	DC with studies	1001€ - 1500€	Other result	3	More than 1 year	Tertiary Education	Tertiary Education	Full- time job
52	Men	1.968	DC with studies	3001€ or more	Gold	5	I was already working	Tertiary Education	Tertiary Education	Full- time job
53	Men	1.969	DC with studies	2000€ - 3000€	Other result	3	I was already working	Secondary Education	Tertiary Education	Temporary job
54	Men	1.968	DC with studies	2000€ - 3000€	Other result	4	Less than 1 year	Secondary Education	Tertiary Education	Full- time job
55	Men	1.964	DC with studies	601€ - 1000€	Diploma	Very bad	More than 1 year	Tertiary Education	Tertiary Education	#¡NULO!
56	Men	1.960	DC with studies	3001€ or more	Diploma	3	More than 1 year	Tertiary Education	Tertiary Education	Entrepreneur
57	Men	1.968	DC with studies	3001€ or more	Gold	5	I was already working	Tertiary Education	Tertiary Education	Full- time job
58	Men	1.968	DC with studies	2000€ - 3000€	Other result	3	Less than 1 year	Secondary Education	Tertiary Education	Full- time job
59	Men	1.968	DC with studies	1501€ - 2000€	Other result	Very bad	More than 1 year	Secondary Education	Secondary Education	Full- time job
60	Men	1.963	DC with studies	1501€ - 2000€	Other result	Very well	I was already working	Professional Training	Tertiary Education	Full- time job
61	Men	1.960	DC with studies	3001€ or more	Other result	3	Less than 1 year	Tertiary Education	Tertiary Education	Profesional
62	Men	1.967	DC with studies	3001€ or more	Other result	5	More than 1 year	Secondary Education	Tertiary Education	Full- time job
63	Men	1.971	DC with studies	3001€ or more	Silver	5	I was already working	Secondary Education	Tertiary Education	Entrepreneur
64	Men	1.963	DC with studies	2000€ - 3000€	Diploma	Very well	I was already working	Tertiary Education	Tertiary Education	Full- time job
65	Men	1.965	DC with studies	2000€ - 3000€	Diploma	Very well	I was already working	Tertiary Education	Tertiary Education	Full- time job
66	Men	1.969	DC with studies	3001€ or more	Other result	Very bad	Less than 1 year	Secondary Education	Tertiary Education	Full- time job
67	Men	1.967	DC with studies	1001€ - 1500€	Gold	Very well	I was already working	Tertiary Education	Tertiary Education	Full- time job
68	Men	1.970	DC with studies	1501€ - 2000€	Diploma	Very bad	Less than 1 year	Secondary Education	Tertiary Education	Full- time job
69	Men	1.968	DC with studies	1001€ - 1500€	Other result	4	Less than 1 year	Secondary Education	Secondary Education	Full- time job
70	Men	1.967	DC with studies	1501€ - 2000€	Other result	3	I was already working	Tertiary Education	Tertiary Education	Full- time job
71	Men	1.969	DC with studies	2000€ - 3000€	Gold	4	Less than 1 year	Secondary Education	Secondary Education	Full- time job
72	Men	1.968	DC with studies	1501€ - 2000€	Other result	5	I was already working	Tertiary Education	Tertiary Education	Full- time job
73	Men	1.971	DC with studies	3001€ or more	Diploma	Very well	Less than 1 year	Secondary Education	Tertiary Education	Full- time job
74	Men	1.969	DC with studies	1501€ - 2000€	Other result	4	More than 1 year	Professional Training	Professional Training	Entrepreneur
75	Men	1.968	DC with studies	2000€ - 3000€	Other result	3	I was already working	Tertiary Education	Tertiary Education	Full- time job
76	Men	1.965	DC with studies	#¡NULO!	Diploma	Very well	Less than 1 year	Tertiary Education	Tertiary Education	Full- time job
77	Men	#¡NULO!	DC with studies	3001€ or more	Other result	Very bad	I was already working	Tertiary Education	Tertiary Education	Full- time job
78	Men	1.968	DC with studies	2000€ - 3000€	Other result	3	Less than 1 year	Secondary Education	Tertiary Education	Full- time job
79	Men	1.972	DC with studies	3001€ or more	Bronze	#¡NULO!	#¡NULO!	Secondary Education	Secondary Education	#¡NULO!
80	Men	1.964	DC with studies	601€ - 1000€	Silver	Very bad	I was already working	Tertiary Education	Tertiary Education	Profesional
81	Men	1.966	DC with studies	3001€ or more	Silver	4	Less than 1 year	Tertiary Education	Tertiary Education	Full- time job
82	Men	1.974	DC with studies	3001€ or more	Gold	3	I was already working	Tertiary Education	Tertiary Education	Full- time job
83	Men	1.966	DC with studies	1501€ - 2000€	Silver	5	Less than 1 year	Secondary Education	Secondary Education	Full- time job
84	Men	1.966	DC with studies	3001€ or more	Silver	#¡NULO!	I was already working	Tertiary Education	Tertiary Education	Full- time job
85	Men	1.961	DC with studies	601€ - 1000€	Diploma	3	Less than 1 year	Primary Education	Primary Education	Self-employed
86	Men	1.972	DC with studies	1501€ - 2000€	Gold	#¡NULO!	#¡NULO!	Secondary Education	Tertiary Education	#¡NULO!
87	Men	1.969	DC with studies	3001€ or more	Bronze	2	Less than 1 year	Tertiary Education	Tertiary Education	Full- time job
88	Men	1.975	DC with studies	3001€ or more	Bronze	#¡NULO!	#¡NULO!	Professional Training	Tertiary Education	Full- time job
89	Men	1.972	DC with studies	2000€ - 3000€	Other result	Very well	I was already working	Tertiary Education	Tertiary Education	Full- time job
90	Men	1.970	DC with studies	2000€ - 3000€	Other result	Very well	I was already working	Secondary Education	Secondary Education	Full- time job
91	Men	1.966	DC with studies	2000€ - 3000€	Other result	3	Less than 1 year	Secondary Education	Tertiary Education	Full- time job
92	Men	1.969	DC with studies	3001€ or more	Other result	Very well	Less than 1 year	Tertiary Education	Tertiary Education	Full- time job
93	Men	1.966	DC with studies	2000€ - 3000€	Other result	Very well	Less than 1 year	Tertiary Education	Tertiary Education	Entrepreneur
94	Men	1.970	DC with studies	2000€ - 3000€	Other result	3	I was already working	Tertiary Education	Tertiary Education	Full- time job
95	Men	1.969	DC with studies	2000€ - 3000€	Other result	3	I was already working	Secondary Education	Tertiary Education	Temporary job
96	Men	1.970	DC with studies	2000€ - 3000€	Other result	3	Less than 1 year	Secondary Education	Tertiary Education	Full- time job
97	Men	1.968	DC with studies	2000€ - 3000€	Other result	5	I was already working	Tertiary Education	Tertiary Education	Full- time job
98	Men	1.979	DC with studies	2000€ - 3000€	Other result	2	I was already working	Secondary Education	Tertiary Education	Full- time job
99	Men	1.978	DC with studies	601€ - 1000€	Other result	#¡NULO!	#¡NULO!	Professional Training	Tertiary Education	#¡NULO!
100	Men	1.966	DC with studies	3001€ or more	Other result	Very well	I was already working	Tertiary Education	Tertiary Education	Full- time job
101	Men	1.975	DC with studies	1001€ - 1500€	Diploma	Very well	Less than 1 year	Tertiary Education	Tertiary Education	Self-employed
102	Men	1.977	DC with studies	1501€ - 2000€	Other result	Very well	I was already working	Tertiary Education	Tertiary Education	Full- time job
103	Men	1.977	DC with studies	2000€ - 3000€	Other result	Very well	I was already working	Tertiary Education	Tertiary Education	Full- time job
104	Men	1.965	DC with studies	2000€ - 3000€	Diploma	Very bad	More than 1 year	Tertiary Education	Tertiary Education	Self-employed
105	Men	1.977	DC with studies	2000€ - 3000€	Other result	4	I was already working	Primary Education	Tertiary Education	Full- time job
106	Men	1.978	DC with studies	1001€ - 1500€	Other result	2	More than 1 year	Tertiary Education	Tertiary Education	Full- time job
107	Men	1.975	DC with studies	3001€ or more	Diploma	#¡NULO!	#¡NULO!	Secondary Education	Secondary Education	Self-employed
108	Men	1.976	DC with studies	1501€ - 2000€	Other result	4	I was already working	Tertiary Education	Tertiary Education	Entrepreneur
109	Men	1.970	DC with studies	1501€ - 2000€	Diploma	Very well	Less than 1 year	Tertiary Education	Tertiary Education	Full- time job
110	Men	1.978	DC with studies	3001€ or more	Other result	#¡NULO!	I was already working	Secondary Education	Tertiary Education	Full- time job

111	Men	1.976	DC with studies	1501€ - 2000€	Other result	4	I was already working	Tertiary Education	Tertiary Education	Entrepreneur
112	Men	1.978	DC with studies	Up to 600€	Other result	3	More than 1 year	Tertiary Education	Tertiary Education	Part-time job
113	Men	1.975	DC with studies	2000€ - 3000€	Other result	#¡NULO!	#¡NULO!	Secondary Education	Secondary Education	Temporary job
114	Men	1.970	DC with studies	3001€ or more	Other result	5	I was already working	Tertiary Education	Tertiary Education	Profesional
115	Men	1.971	DC with studies	3001€ or more	Bronze	4	Less than 1 year	Tertiary Education	Tertiary Education	Full- time job
116	Men	1.971	DC with studies	3001€ or more	Other result	Very well	Less than 1 year	Tertiary Education	Tertiary Education	Full- time job
117	Men	1.972	DC with studies	3001€ or more	Bronze	Very well	Less than 1 year	Tertiary Education	Tertiary Education	Full- time job
118	Men	1.977	DC with studies	2000€ - 3000€	Other result	5	I was already working	Tertiary Education	Tertiary Education	Full- time job
119	Men	1.978	DC with studies	1501€ - 2000€	Other result	4	I was already working	Secondary Education	Secondary Education	Full- time job
120	Men	1.965	DC with work	601€ - 1000€	Diploma	Very bad	Less than 1 year	Professional Training	Tertiary Education	Part-time job
121	Men	1.968	DC with work	1001€ - 1500€	Other result	4	I was already working	Professional Training	Professional Training	Full- time job
122	Men	1.965	DC with work	1001€ - 1500€	Diploma	3	I was already working	Secondary Education	Secondary Education	Full- time job
123	Men	1.965	DC with work	1001€ - 1500€	Diploma	#¡NULO!	#¡NULO!	Primary Education	Primary Education	Full- time job
124	Men	1.956	DC with work	1501€ - 2000€	Diploma	Very well	I was already working	Secondary Education	Secondary Education	Full- time job
125	Men	1.962	DC with work	2000€ - 3000€	Diploma	2	I was already working	Secondary Education	Tertiary Education	Part-time job
126	Men	1.962	DC with work	3001€ or more	Diploma	Very well	I was already working	Professional Training	Professional Training	Part-time job
127	Men	1.963	DC with work	3001€ or more	Silver	5	I was already working	Tertiary Education	Tertiary Education	Full- time job
128	Men	1.964	DC with work	2000€ - 3000€	Diploma	#¡NULO!	#¡NULO!	Primary Education	Primary Education	Full- time job
129	Men	1.960	DC with work	1001€ - 1500€	Other result	4	I was already working	Secondary Education	Secondary Education	Self-employed
130	Men	1.964	DC with work	2000€ - 3000€	Other result	4	Less than 1 year	Secondary Education	Secondary Education	Full- time job
131	Men	1.958	DC with work	Up to 600€	Diploma	4	#¡NULO!	Secondary Education	Secondary Education	Entrepreneur
132	Men	1.965	DC with work	2000€ - 3000€	Diploma	Very well	I was already working	Tertiary Education	Tertiary Education	Full- time job
133	Women	1.967	Solely devoted to sport	1501€ - 2000€	Diploma	2	Less than 1 year	Tertiary Education	Tertiary Education	Part-time job
134	Women	1.973	Solely devoted to sport	601€ - 1000€	Gold	4	More than 1 year	Secondary Education	Secondary Education	Full- time job
135	Women	1.966	Solely devoted to sport	601€ - 1000€	Other result	2	Less than 1 year	Secondary Education	Secondary Education	Full- time job
136	Women	1.967	Solely devoted to sport	1001€ - 1500€	Other result	4	More than 1 year	Secondary Education	Tertiary Education	Self-employed
137	Women	1.976	Solely devoted to sport	1001€ - 1500€	Silver	3	Less than 1 year	Primary Education	Secondary Education	Part-time job
138	Women	1.969	Solely devoted to sport	601€ - 1000€	Other result	5	Less than 1 year	Secondary Education	Secondary Education	Full- time job
139	Women	1.964	Solely devoted to sport	1501€ - 2000€	Diploma	4	I was already working	Tertiary Education	Tertiary Education	Full- time job
140	Women	1.969	Solely devoted to sport	#¡NULO!	#¡NULO!	3	#¡NULO!	Primary Education	Primary Education	#¡NULO!
141	Women	1.971	Solely devoted to sport	1001€ - 1500€	Diploma	4	Less than 1 year	Secondary Education	Secondary Education	Entrepreneur
142	Women	1.968	Solely devoted to sport	1501€ - 2000€	Diploma	5	I was already working	Tertiary Education	Tertiary Education	Full- time job
143	Women	1.974	Solely devoted to sport	601€ - 1000€	Other result	4	Less than 1 year	Primary Education	Primary Education	Full- time job
144	Women	1.971	Solely devoted to sport	1001€ - 1500€	Diploma	3	I was already working	Secondary Education	Tertiary Education	Part-time job
145	Women	1.963	Solely devoted to sport	1001€ - 1500€	Other result	3	Less than 1 year	Primary Education	Primary Education	Part-time job
146	Women	1.971	Solely devoted to sport	1501€ - 2000€	Diploma	3	Less than 1 year	Secondary Education	Secondary Education	Full- time job
147	Women	1.969	Solely devoted to sport	1501€ - 2000€	Silver	3	I was already working	Secondary Education	Tertiary Education	Full- time job
148	Women	1.975	Solely devoted to sport	1001€ - 1500€	Other result	2	I was already working	Tertiary Education	Tertiary Education	Part-time job
149	Women	1.978	Solely devoted to sport	601€ - 1000€	Diploma	Very bad	More than 1 year	Primary Education	Secondary Education	Part-time job
150	Women	1.974	Solely devoted to sport	601€ - 1000€	Other result	4	Less than 1 year	Primary Education	Primary Education	Full- time job
151	Women	1.969	Solely devoted to sport	1001€ - 1500€	Other result	Very bad	More than 1 year	Secondary Education	Secondary Education	Part-time job
152	Women	1.966	Solely devoted to sport	3001€ or more	#¡NULO!	Very well	Less than 1 year	Secondary Education	Secondary Education	Full- time job
153	Women	1.972	DC with studies	2000€ - 3000€	Other result	4	More than 1 year	Secondary Education	Tertiary Education	Full- time job
154	Women	1.969	DC with studies	1001€ - 1500€	Diploma	Very well	I was already working	Professional Training	Professional Training	Full- time job
155	Women	1.963	DC with studies	3001€ or more	Diploma	Very well	I was already working	Tertiary Education	Tertiary Education	#¡NULO!
156	Women	1.973	DC with studies	1501€ - 2000€	Gold	Very well	I was already working	Secondary Education	Tertiary Education	Full- time job
157	Women	1.977	DC with studies	601€ - 1000€	Other result	#¡NULO!	#¡NULO!	Primary Education	Tertiary Education	Temporary job
158	Women	1.964	DC with studies	3001€ or more	Diploma	4	I was already working	Tertiary Education	Tertiary Education	Entrepreneur
159	Women	1.963	DC with studies	1001€ - 1500€	Other result	Very well	Less than 1 year	Tertiary Education	Tertiary Education	Full- time job
160	Women	1.960	DC with studies	1501€ - 2000€	Diploma	Very well	Less than 1 year	Tertiary Education	Tertiary Education	Full- time job
161	Women	1.968	DC with studies	1001€ - 1500€	Other result	5	Less than 1 year	Tertiary Education	Tertiary Education	Full- time job
162	Women	1.964	DC with studies	1501€ - 2000€	Gold	5	I was already working	Secondary Education	Secondary Education	Full- time job
163	Women	1.966	DC with studies	1001€ - 1500€	Other result	3	I was already working	Tertiary Education	Tertiary Education	Full- time job
164	Women	1.971	DC with studies	2000€ - 3000€	Other result	5	More than 1 year	Tertiary Education	Tertiary Education	Full- time job
165	Women	1.974	DC with studies	1501€ - 2000€	Other result	5	More than 1 year	Secondary Education	Tertiary Education	Full- time job
166	Women	1.966	DC with studies	2000€ - 3000€	Diploma	Very well	Less than 1 year	Tertiary Education	Tertiary Education	Entrepreneur
167	Women	1.969	DC with studies	601€ - 1000€	Other result	4	Less than 1 year	Professional Training	Professional Training	Part-time job
168	Women	1.976	DC with studies	1001€ - 1500€	Other result	4	Less than 1 year	Primary Education	Secondary Education	Self-employed
169	Women	1.967	DC with studies	#¡NULO!	Diploma	2	More than 1 year	Secondary Education	Tertiary Education	#¡NULO!
170	Women	1.967	DC with studies	Up to 600€	Gold	2	Less than 1 year	Tertiary Education	Tertiary Education	Part-time job
171	Women	1.970	DC with studies	2000€ - 3000€	Other result	5	Less than 1 year	Tertiary Education	Tertiary Education	Profesional
172	Women	1.970	DC with studies	#¡NULO!	#¡NULO!	#¡NULO!	More than 1 year	Secondary Education	Tertiary Education	#¡NULO!
173	Women	1.970	DC with studies	1001€ - 1500€	Gold	Very well	More than 1 year	Professional Training	Tertiary Education	Entrepreneur
174	Women	1.968	DC with studies	1001€ - 1500€	Gold	4	I was already working	Secondary Education	Secondary Education	Full- time job
175	Women	1.972	DC with studies	601€ - 1000€	Other result	5	More than 1 year	Secondary Education	Tertiary Education	Part-time job
176	Women	1.971	DC with studies	1501€ - 2000€	Diploma	3	Less than 1 year	Secondary Education	Tertiary Education	Self-employed
177	Women	1.969	DC with studies	1501€ - 2000€	Other result	Very bad	More than 1 year	Tertiary Education	Tertiary Education	Full- time job
178	Women	1.971	DC with studies	601€ - 1000€	#¡NULO!	#¡NULO!	I was already working	#¡NULO!	#¡NULO!	Self-employed
179	Women	1.970	DC with studies	2000€ - 3000€	Other result	4	Less than 1 year	Secondary Education	Tertiary Education	Full- time job
180	Women	1.958	DC with studies	1001€ - 1500€	Other result	2	I was already working	Secondary Education	Tertiary Education	Temporary job
181	Women	1.973	DC with studies	1001€ - 1500€	Silver	3	More than 1 year	Secondary Education	Tertiary Education	Profesional
182	Women	1.973	DC with studies	3001€ or more	Gold	4	Less than 1 year	Secondary Education	Tertiary Education	Profesional
183	Women	1.966	DC with studies	#¡NULO!	Diploma	Very well	More than 1 year	Tertiary Education	Tertiary Education	Full- time job
184	Women	1.975	DC with studies	1501€ - 2000€	Diploma	3	Less than 1 year	Secondary Education	Tertiary Education	Full- time job
185	Women	1.973	DC with studies	1501€ - 2000€	Gold	Very well	I was already working	Secondary Education	Tertiary Education	Full- time job
186	Women	1.973	DC with studies	3001€ or more	Diploma	3	Less than 1 year	Tertiary Education	Tertiary Education	Profesional
187	Women	1.972	DC with studies	2000€ - 3000€	Other result	5	Less than 1 year	Tertiary Education	Tertiary Education	Entrepreneur
188	Women	1.970	DC with studies	1001€ - 1500€	Other result	2	Less than 1 year	Tertiary Education	Tertiary Education	Part-time job
189	Women	1.976	DC with studies	1501€ - 2000€	Other result	2	Less than 1 year	Secondary Education	Tertiary Education	Full- time job
190	Women	1.972	DC with studies	601€ - 1000€	Other result	5	More than 1 year	Secondary Education	Tertiary Education	Part-time job
191	Women	1.968	DC with studies	1001€ - 1500€	Gold	4	I was already working	Secondary Education	Secondary Education	Full- time job
192	Women	1.981	DC with studies	1001€ - 1500€	Diploma	5	More than 1 year	Primary Education	Tertiary Education	#¡NULO!
193	Women	1.981	DC with studies	1501€ - 2000€	Gold	3	More than 1 year	Primary Education	Tertiary Education	Entrepreneur
194	Women	1.969	DC with studies	601€ - 1000€	Other result	5	Less than 1 year	Professional Training	Professional Training	Part-time job
195	Women	1.968	DC with studies	1001€ - 1500€	Other result	5	Less than 1 year	Tertiary Education	Tertiary Education	Full- time job
196	Women	1.979	DC with studies	1501€ - 2000€	Other result	5	Less than 1 year	Secondary Education	Tertiary Education	Self-employed
197	Women	1.975	DC with studies	1001€ - 1500€	Other result	#¡NULO!	I was already working	Tertiary Education	Tertiary Education	Full- time job
198	Women	1.978	DC with studies	2000€ - 3000€	Diploma	Very well	More than 1 year	Tertiary Education	Tertiary Education	Self-employed
199	Women	1.982	DC with studies	#¡NULO!	Other result	#¡NULO!	#¡NULO!	Secondary Education	Tertiary Education	#¡NULO!
200	Women	1.978	DC with studies	#¡NULO!	Diploma	#¡NULO!	#¡NULO!	Professional Training	Professional Training	#¡NULO!
201	Women	1.983	DC with studies	1001€ - 1500€	Other result	3	More than 1 year	Primary Education	Tertiary Education	Part-time job
202	Women	1.968	DC with studies	1501€ - 2000€	Diploma	5	Less than 1 year	Tertiary Education	Tertiary Education	Full- time job
203	Women	1.973	DC with studies	2000€ - 3000€	Diploma	3	Less than 1 year	Tertiary Education	Tertiary Education	Part-time job
204	Women	1.969	DC with studies	1501€ - 2000€	Diploma	3	I was already working	Tertiary Education	Tertiary Education	#¡NULO!
205	Women	1.977	DC with studies	1501€ - 2000€	Other result	4	Less than 1 year	Tertiary Education	Tertiary Education	Full- time job
206	Women	1.975	DC with studies	2000€ - 3000€	Diploma	3	Less than 1 year	Tertiary Education	Tertiary Education	Full- time job
207	Women	1.974	DC with studies	1001€ - 1500€	Other result	4	I was already working	Tertiary Education	Tertiary Education	Part-time job
208	Women	1.982	DC with studies	1001€ - 1500€	Diploma	3	More than 1 year	Secondary Education	Tertiary Education	Full- time job
209	Women	1.973	DC with studies	Up to 600€	Diploma	5	Less than 1 year	Tertiary Education	Tertiary Education	Part-time job
210	Women	1.978	DC with studies	3001€ or more	Diploma	4	More than 1 year	Tertiary Education	Tertiary Education	Self-employed
211	Women	1.978	DC with studies	2000€ - 3000€	Diploma	5	I was already working	Tertiary Education	Tertiary Education	Full- time job
212	Women	1.975	DC with studies	1501€ - 2000€	Diploma	Very well	I was already working	Tertiary Education	Tertiary Education	Full- time job
213	Women	1.980	DC with studies	#¡NULO!	Other result	3	Less than 1 year	Secondary Education	Secondary Education	Temporary job
214	Women	1.980	DC with studies	1501€ - 2000€	Other result	Very well	I was already working	Tertiary Education	Tertiary Education	Full- time job
215	Women	1.983	DC with studies	1001€ - 1500€	Other result	3	#¡NULO!	Primary Education	Tertiary Education	Part-time job
216	Women	1.976	DC with studies	1001€ - 1500€	Diploma	Very bad	Less than 1 year	Secondary Education	Secondary Education	Full- time job
217	Women	1.979	DC with studies	1501€ - 2000€	Diploma	#¡NULO!	I was already working	Tertiary Education	Tertiary Education	Self-employed
218	Women	1.980	DC with studies	2000€ - 3000€	Other result	4	I was already working	Secondary Education	Tertiary Education	Full- time job
219	Women	1.976	DC with studies	#¡NULO!	Other result	#¡NULO!	#¡NULO!	Tertiary Education	Tertiary Education	#¡NULO!
220	Women	1.957	DC with work	1501€ - 2000€	Gold	4	I was already working	Tertiary Education	Tertiary Education	Full- time job
221	Women	1.969	DC with work	1501€ - 2000€	Other result	Very well	I was already working	Primary Education	Tertiary Education	Full- time job

222	Women	1.949	DC with work	1501€ - 2000€	Other result	Very well	I was already working	Tertiary Education	Tertiary Education	Profesional
223	Women	1.963	DC with work	601€ - 1000€	Other result	4	I was already working	Secondary Education	Secondary Education	Full- time job
224	Women	1.969	DC with work	1501€ - 2000€	Other result	3	I was already working	Secondary Education	Tertiary Education	#¡NULO!
225	Women	1.962	DC with work	2000€ - 3000€	Other result	#¡NULO!	#¡NULO!	Professional Training	Professional Training	Full- time job
226	Women	1.969	DC with work	1001€ - 1500€	Other result	Very bad	More than 1 year	Tertiary Education	Tertiary Education	Self-employed
227	Women	1.963	DC with work	1501€ - 2000€	Other result	5	I was already working	Tertiary Education	Tertiary Education	Profesional
228	Women	1.975	DC with work	1001€ - 1500€	Diploma	4	I was already working	Secondary Education	Secondary Education	Temporary job
